# Modulation of iron homeostasis in macrophages by bacterial intracellular pathogens

**DOI:** 10.1186/1471-2180-10-64

**Published:** 2010-02-25

**Authors:** Xin Pan, Batcha Tamilselvam, Eric J Hansen, Simon Daefler

**Affiliations:** 1 Mount Sinai School of Medicine, One Gustave Levy Place, New York, NY 10570, USA; 2 UTSW Medical Center at Dallas, Department of Microbiology, Dallas, TX, USA

## Abstract

**Background:**

Intracellular bacterial pathogens depend on acquisition of iron for their success as pathogens. The host cell requires iron as an essential component for cellular functions that include innate immune defense mechanisms. The transferrin receptor TfR1 plays an important part for delivering iron to the host cell during infection. Its expression can be modulated by infection, but its essentiality for bacterial intracellular survival has not been directly investigated.

**Results:**

We identified two distinct iron-handling scenarios for two different bacterial pathogens. *Francisella tularensis *drives an active iron acquisition program via the TfR1 pathway program with induction of ferrireductase (Steap3), iron membrane transporter Dmt1, and iron regulatory proteins IRP1 and IRP2, which is associated with a sustained increase of the labile iron pool inside the macrophage. Expression of TfR1 is critical for *Francisella's *intracellular proliferation. This contrasts with infection of macrophages by wild-type *Salmonella typhimurium*, which does not require expression of TfR1 for successful intracellular survival. Macrophages infected with *Salmonella *lack significant induction of Dmt1, Steap3, and IRP1, and maintain their labile iron pool at normal levels.

**Conclusion:**

The distinction between two different phenotypes of iron utilization by intracellular pathogens will allow further characterization and understanding of host-cell iron metabolism and its modulation by intracellular bacteria.

## Background

Iron is required by a wide variety of intracellular bacterial pathogens to achieve full virulence. Deprivation of iron in-vivo and in-vitro severely reduces the pathogenicity of *Mycobacterium tuberculosis, Coxiella burnettii, Legionella pneumophila*, and *Salmonella typhimurium *[1-4]. Attempts to withhold iron by sequestering free iron during infection is a major defense strategy used by many species [5]. Inflammatory signaling cascades during infection lead to a reduction in available free iron and sequestration of iron in the reticuloendothelial system (RES) [6]. On the other hand, iron is needed by host cells for cellular functions and first line defense mechanisms [7]. Iron homeostasis also affects macrophage and lymphocyte effector pathways of the innate and adaptive immune response [6,8].

Iron homeostasis in the macrophage is determined by uptake processes through lactoferrin, transferrin, divalent metal transporter (DMT-1), phagocytosis of senescent erythrocytes, and by export through ferroportin (Fpn1) [8]. Transferrin and its receptor (TfR1) play an important role during infection of macrophages with bacterial pathogens that prefer an intracellular lifestyle. Expression of TfR1 can in turn be modulated by bacterial infections [9]. Intracellular bacteria such as *Mycobacterium tuberculosis *and *Ehrlichia *[10,11] actively recruit TfR1 to the bacterium-containing vacuole. However, the requirement of TfR1 for bacterial pathogenesis has not been directly addressed.

We sought here to determine if iron delivery through the transferrin receptor (TfR1) is essential for the success of two intracellular pathogens with differing intracellular life-styles, *Salmonella typhimurium *and *Francisella tularensis*. *Salmonella typhimurium *represents a well-characterized model intracellular pathogen, which causes typhoid fever in the mouse. *Salmonella *uncouples from the phagolysosomal pathway in macrophages and remains in a protected intracellular niche inside a vacuole [12]. The *Salmonella*-containing vacuole (SCV) interacts with multiple endocytic pathways and avoids its fusion with acidic lysosomes. This is similar to infection with *Chlamydia*, *Legionella*, and *Mycobacteriae*. In contrast, *Francisella tularensis*, causative agent of tularemia and considered a category A biothreat because of its high infectivity and high case-fatality rate when untreated, enters the macrophage in a vesicle, but escapes from its enclosure into the cytosol after lysis of its vesicle within sixty minutes after entry into the host cell [13]. Both *Francisella *and *Salmonella *require iron for successful intracellular proliferation [14]. A *Francisella *operon, *figABCD*, has recently been described as being involved in iron acquisition [15,14]. Recent studies from two groups using random transposon mutagenesis of either *F. tularensis *LVS [16] or *F. novicida *[17] showed that insertions into the *figA, figB, figC*, or *feoB *genes caused reduced virulence of these mutants. While transposon insertions may cause polar effects on downstream genes, these data strongly suggest that expression of these particular gene products is essential for full virulence of *Francisella *species. In addition, expression of certain *F.tularenis *virulence genes is clearly regulated by iron availability [14,18].

After exposure to just a few aerosolized *Francisella*, serum iron decreases very rapidly [19]. Bacteria counteract the host's withholding of iron by secretion of iron chelators, which are termed siderophores, or by directly interacting with host iron-binding proteins [20-22]. The *Francisella figABCDEF *gene cluster (also referred to as *fslABCDEF *[23]) encodes such a siderophore, which belongs to the polycarboxylate family such as produced by *Rhizopus *species [15,14].

All these studies suggest that a delicate balance of the iron available for bacteria and for host cell metabolism and defense strategies has to be achieved during infection. On the bacterial side, many operons responsible for iron acquisition and scavenging have been described. However, much less is known how the host cell modulates its iron homeostasis and how pathogens might actively influence such homeostasis.

## Results

### Transferrin receptor is required for Francisella intracellular proliferation but not for Salmonella

**Figure 1:**
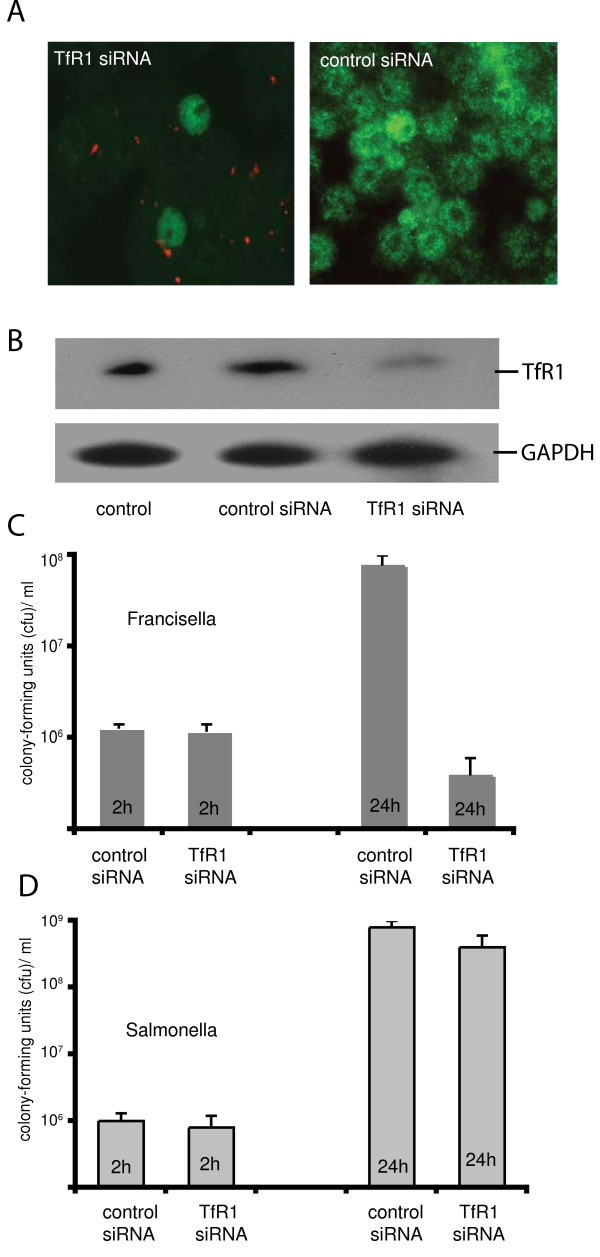
***Francisella*, but not *Salmonella *requires TfR1 for proliferation inside macrophages**. A. RAW264.7 macrophages were transfected with siRNA (coupled to Alexa Fluor 555, red fluorescence) specific for TfR1 or as control with random siRNA (no red fluorescence). After 48 h cells were fixed and processed for immunofluorescence with a mouse anti-TfR1 antibody followed by an Alexa488 conjugated goat-anti-mouse IgG (green fluorescence). Overlay of both fluorescence channels is shown. B. Proteins were solubilized from transfected and infected cells as above, separated on a 9% SDS-PAGE, transferred to Westran membranes, and immunoblotted with antiserum to TfR1. Visualization was by chemiluminescence C. RAW264.7 macrophages were transfected with TfR1-siRNA or with random siRNA (control). 48 h cells after transfection cells were infected with *Francisella *for 2 h or 24 h. The number of intracellular bacteria was obtained by plating a lysate of the host cells on chocolate agar plates for colony-forming units (cfus). Means of triplicate experiments +/- 1 standard error of mean are shown. D. RAW264.7 cells were treated as in C and then infected with *Salmonella *for 2 h or 24 h. The number of intracellular bacteria was determined as in C. Means of triplicate experiments +/- 1 standard error of mean are shown.

In order to determine if expression of TfR1 is required for proliferation of *Francisella *and *Salmonella *inside macrophages, siRNA was used to silence the expression of TfR1 in murine macrophages (RAW264.7). Expression of the transferrin receptor was suppressed significantly 48 h after transfection with siRNA as measured by fluorescence microscopy and immunoblotting (Figure 1A and 1B). Our transfection efficiency for siRNA was 63% (+/- 7%), which was determined by counting cells, which had taken up siRNA labeled with the red fluorescence dye Alexa Fluor 555 (Figure 1A). Transfected cells appear to have an almost complete reduction of TfR1 (Figure 1A). Thus, the residual expression of transferrin receptors seen by immunoblot (Figure 1B) is most likely due to non-transfected cells.

Macrophages (RAW264.7) transfected with TfR1-siRNA were infected with *Francisella tularensis subspecies holarctica *vaccine strain (*F. tularensis *LVS) or wild-type *Salmonella typhimurium *(ATTC 14208). *F. tularensis *LVS has been developed from fully virulent type B *Francisella *strains. It is attenuated in humans, but virulent in a mouse model [24]. After two hours of infection, there was no difference in the number of intracellular *Salmonella *(p = 0.91) or *Francisella *(p = 0.89) between non-transfected and transfected macrophages (Figure 1C and 1D). This suggests that expression of TfR1 does not affect bacterial entry processes. *Francisella*, however, failed to proliferate in macrophages in which expression of the transferrin receptor was suppressed (Figure 1C; p = 0.005). The amount of *Francisella *recovered after 24 h most likely represents growth in macrophages which could not be transfected with siRNA. In contrast, intracellular proliferation of *S. typhimurium *was not affected by the lack of TfR1 (Figure 1D; p = 0.89). Addition of lactoferrin - chelated iron (Fe content >0.15% w/w, final lactoferrin concentration of 0.01 mg/ml) as external iron source to macrophages with suppressed TfR1 rescued the proliferation of *Francisella *at intermediate levels (data not shown).

### Spatial relationship of transferrin receptor and Francisella-containing vacuole

Some intracellular pathogens have devised ways to attract transferrin receptors to the intracellular vesicles they reside in [11]. When *Salmonella *enters macrophages, it localizes to an early endosome that is characterized by EEA1 and recruitment of the transferrin receptor (TfR1). As the *Salmonella*-containing vacuole matures and acquires markers of late endosomes (Rab7, Rab9), it also loses TfR1 [25,26].

**Figure 2:**
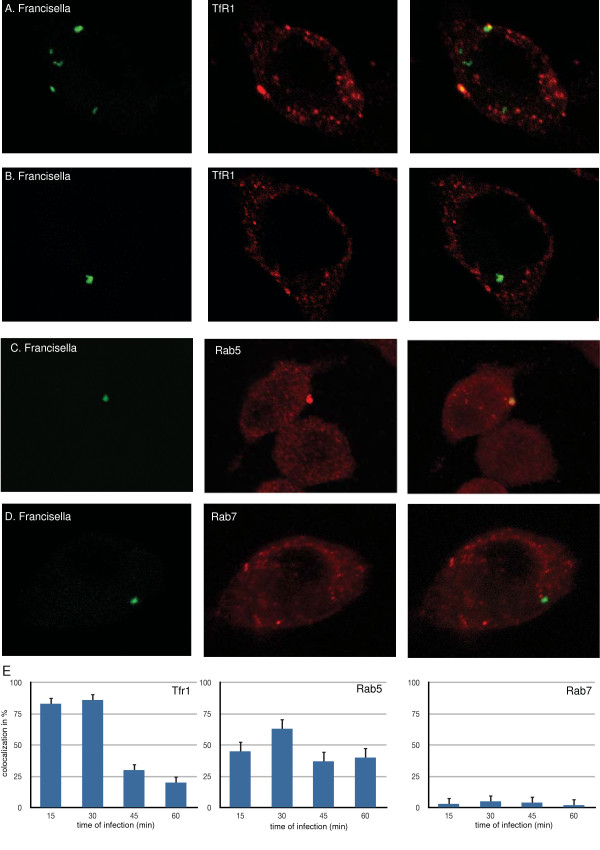
**Transferrin receptor TfR1 and Rab5, but not Rab7, co-localize with *Francisella***. Macrophages (RAW264.7) were infected with *Francisella *that constitutively expressed green fluorescence protein (Gfp). At defined time intervals of infection, cells were fixed and stained with goat anti-TfR1 (A, B), with rabbit anti-Rab5 (C), or goat anti-Rab7 (D), followed by reaction with goat-anti-rabbit or rabbit-anti-goat IgG conjugated to Alexa594 (red fluorescence). Representative confocal images for thirty minutes of infection from twenty *z*-stacks acquired at 0.2 μm intervals are shown for each fluorescence channel, which were then merged using Volocity 4.1 software package (Improvision). E. The colocalization of Francisella with TfR1, Rab5, or Rab7 is described quantitatively for each time point by analyzing 100 infected cells from triplicate independent infection experiments. Means +/- 1 standard error of mean (SEM) are shown.

*Francisella *differs from *Salmonella *by escaping early during infection from its endosomal environment. Since little is known about TfR1 in macrophages infected with *Francisella*, we investigated the role of the transferrin receptor during infection and its relation to the maturation of the *Francisella*-containing vacuole (FCV). Murine macrophages (RAW264.7) were infected with *Francisella *LVS that constitutively expressed Gfp. At defined time intervals, infected cells were fixed and prepared for immunostaining. This demonstrated that early during entry (15 and 30 minutes after infection), there is significant co-localization of FCV and TfR1 (Figure 2A and 2E). As *Francisella *is trafficking away from the cell membrane during the time course of the infection, the co-localization with TfR1 is lost (Figure 2B and 2E; p = 0.88 for comparison of 15 and 30 minutes timepoints, p = 0.006 for 30 and 45 minute timepoints, and p = 0.61 for 45 and 60 minute timepoints (Student's t-test).

Early recycling endosomes are characterized by carrying TfR1, EEA1, and Rab5, while excluding Rab7 unless they are destined for further trafficking along the lysosomal degradation pathway [27]. Macrophages infected with *Francisella *were stained with antisera to Rab5 and Rab7. This demonstrated that *Francisella *very early on at the membrane recruits Rab5 (Figure 2C and 2E; p = 0.09 for 15 and 30 minutes). Colocalization of Francisella and Rab5 decreases over time as Francisella escapes from the vacuole (Figure 2E; p = 0.03 for comparison of 30 and 45 minutes, p = 0.83 for 45 and 60 minutes, Student's t-test). However, there is no co-localization with Rab7-containing vesicles (Figure 2D and 2E; p = 0.88 for comparison of 15 and 30 minutes, p = 0.91 for 30 and 45 minutes, p = 0.89 for 45 and 60 minutes, Student's t-test).

These findings suggest that *Francisella *enters through an early endosome, which is characterized by carrying TfR1 and Rab5. The *Francisella*-containing vacuole does not mature further by acquiring Rab7 and does not retain TfR1. This is most likely due to exit from the vacuole [13] rather than to trafficking to a different vesicle environment with concomitant loss of TfR1.

### Infection of macrophages with Francisella upregulates transferrin receptor

**Figure 3:**
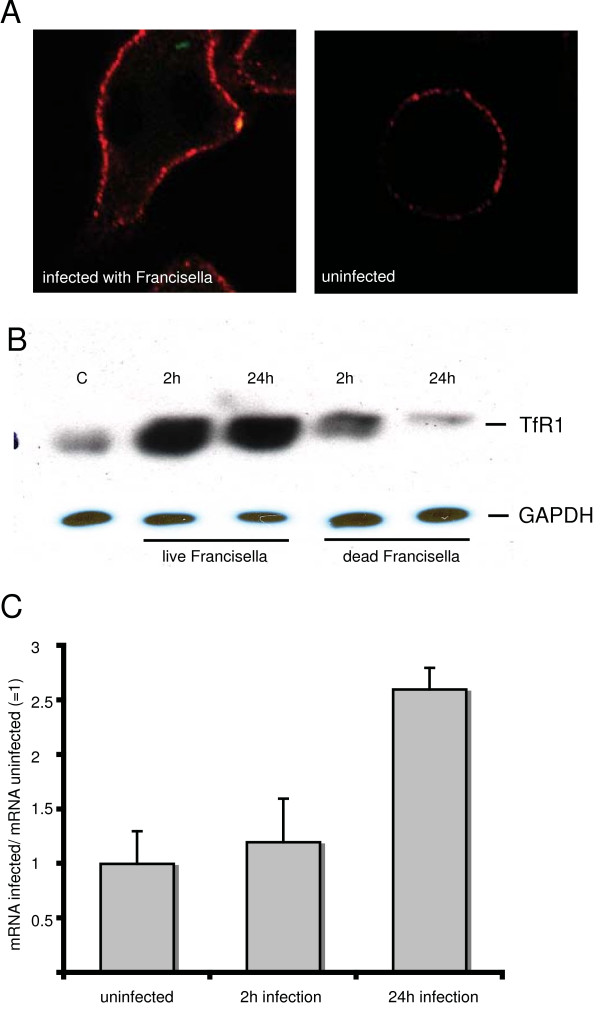
**Infection with *Francisella *increases expression of transferrin receptor**. A. RAW264.7 macrophages were infected with *Francisella *that constitutively expressed Gfp. After 2 h infected cells were fixed and processed for immunofluorescence with a mouse anti-TfR1 antibody followed by an Alexa594 conjugated goat-anti-mouse IgG (red fluorescence). Single confocal planes for merged fluorescence channels are shown. B. RAW264.7 cells were infected with live or formalin-inactivated *Francisella *(dead) for two and twenty-four hours. Immunoblotting of solubilized proteins was done with mouse anti-TfR1 and mouse anti-GAPDH as control. Visualization was by chemiluminescence. C. mRNA levels for TfR1 in RAW264.7 macrophages were determined after 2 or 24 h of infection with *Francisella *by quantitative light cycler PCR; levels are normalized to GAPDH-mRNA levels. Means of n = 6 experiments +/- 1 standard error of mean (SEM) are shown.

Expression of TfR1 remains unchanged during infection with wild-type *Salmonella *[28]. However, when expression of the transferrin receptor in uninfected macrophages was compared by microscopy to the expression in cells infected with *Francisella*, it became evident that *Francisella*-infected macrophages have a higher level of transferrin receptor expression (Figure 3A). This was confirmed by comparing the expression level of the transferrin receptor in *Francisella*-infected macrophages to the level found in uninfected cells by immunblotting at one hour and twenty-four hours after infection (Figure 3B). We also tested the expression level of transferrin receptor in cells, which had taken up formalin-fixed *Francisella*. This did not lead to a comparable upregulation of TfR1 (Figure 3B). Synthesis of the transferrin receptor is mainly regulated at the translational level as a response to the iron level or to other inputs. Indeed, after two hours of infection there was no increase in the mRNA level for Tfr1 as determined by real-time RT-PCR (Figure 3C; p = 0.29). However, after 24 h of infection, the mRNA level for TfR1 had more than doubled (Figure 3C; p = 0.002).

### Increased level of transferrin receptor in infected cells can increase the labile iron pool

An increased TfR1 expression could translate into enhanced transferrin-mediated delivery of iron into the host cell and increased iron availability for *Francisella*. For *Francisella*, this could be accomplished by transferrin directly binding to the bacterial cell surface via a transferrin-binding protein, as has been described for other, mostly extracellular bacteria [20]. Search of the *Francisella *genome did not reveal any homologue to transferrin-binding proteins ((S.Daefler, unpublished observation). We could also experimentally verify that apo-transferrin and holotransferrin do not bind to *Francisella *(data not shown).

We therefore asked if the increased expression of TfR1 correlates with an increase of iron delivery to the host cell. In most cells, uptake of transferrin-bound iron leads to fast delivery into the cytosolic labile iron pool, which can be operationally defined as the cell chelatable pool that includes Fe^2+ ^and Fe^3+ ^associated with ligands such as organic anions, polypeptides, or surface membrane components [29]. The labile iron pool (LIP) composes the metabolically active and regulatory forms of iron [[29,30], Breuer et al., 2007, Int J Biochem Cell Biol]. A sensitive way to measure the labile iron pool without cell disruption is the use of a membrane permeable fluorescent probe such as calcein. Calcein rapidly forms a complex with iron in a 1:1 stoichiometry. This results in quenching of the green fluorescence of calcein. When cells are loaded so that there is a minor excess of free fluorescent calcein, an increase in the LIP will result in a decrease of the fluorescence signal [31], whereas the total cell-associated LIP can be determined after dequenching of the fluorescence signal with a cell-permeant Fe-chelator [29].

**Figure 4:**
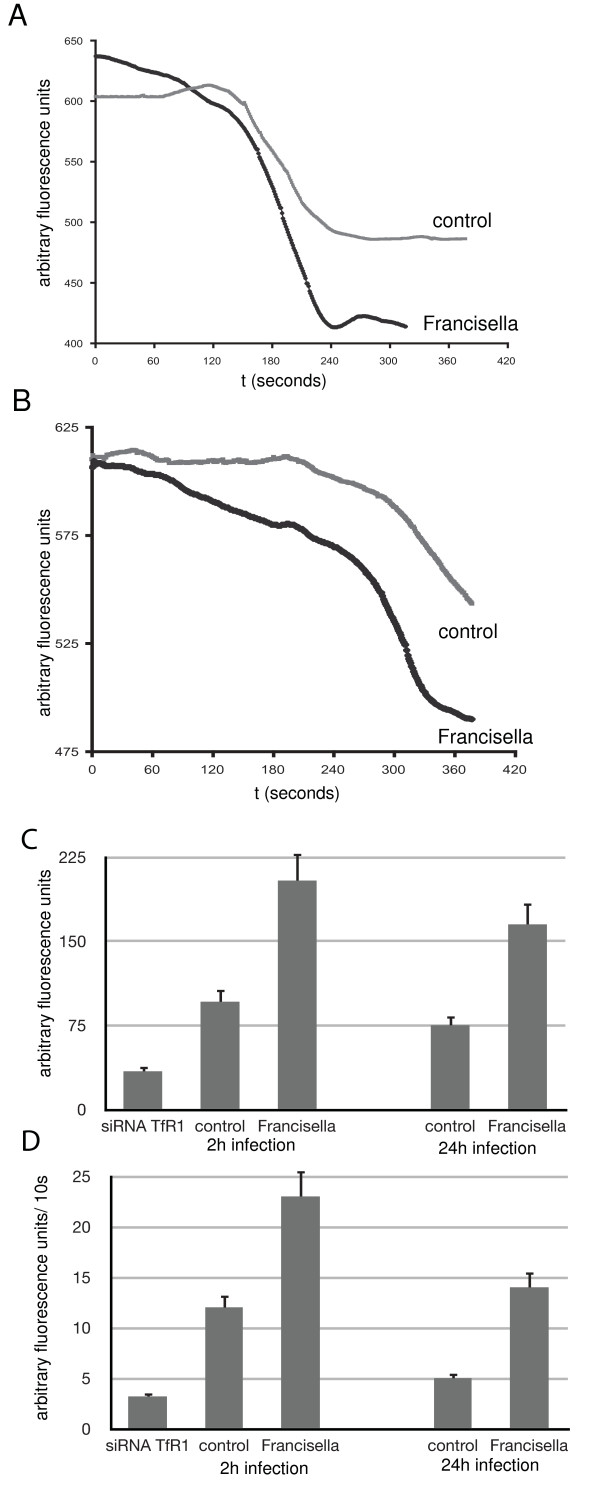
**Transferrin-mediated delivery of iron increases the labile iron pool in *Francisella*-infected cells more efficiently than in uninfected cells**. RAW macrophages were infected with *Francisella *LVS for 2 h (A) or 24 h (B) or left uninfected (control) and then loaded with Calcein-AM. The cell suspension was maintained at 37°C in a fluorometer. After stabilization of the fluorescence signal, holo-transferrin was added to the solution (t = 0) and the fluorescence signal recorded at one-second intervals. A decrease in the fluorescence indicates chelation of incoming iron with calcein, the amount of which is proportional to the slope and amplitude of the fluorescence signal. Results of triplicate measurements from triplicate experiments (n = 9) as described in A and B were analyzed for total amount of iron acquired as measured by arbitrary fluorescence units (C) and velocity of iron acquisition as measured by the change of fluorescence over time (D). Total iron and rate of iron uptake was also analyzed for macrophages whose TfR1 expression was suppressed by siRNA (siRNA TfR1 in Figure 4C and 4D). Measurements were made 24 h after transfection of uninfected macrophages (RAW264.7) with siRNA. All Values are given as means +/- 1 standard error of mean (SEM).

Macrophages were infected with *Francisella *for two and twenty-four hours or left uninfected as control. After loading with calcein, cells were exposed to holotransferrin as delivery vehicle for iron while the fluorescence signal was measured. In macrophages infected with *Francisella*, there is a rapid iron uptake as determined by the slope of the fluorescence quenching, which is steeper than in the control sample (uninfected cells) (Figure 4A, 4B, and 4D; p = 0.0002 for 2 h infection, p = 0.002 for 24 h infection, Student's t-test). Infected macrophages also appear to at least transiently increase the LIP more than uninfected cells, as evidenced by the amplitude of fluorescence quenching (Figure 4A, 4B, and 4C; p = 0.003 for 2 h infection, p = 0.001 for 24 h infection, Student's t-test). This observation is consistent with an increased number of TfRs on the cell surface, allowing an increased uptake at a faster rate of iron into the cell. The iron measured here is at least temporarily available as soluble iron and should thus be readily available for uptake by *Francisella*. In contrast, when we measured the LIP of macrophages whose TfR1 expression has been suppressed by siRNA, we found a decreased LIP (Figure 4C; p = 0.001) and a decreased rate of iron uptake (Figure 4D; p = 0.001).

### Labile iron pool during infection with Francisella or Salmonella

**Figure 5:**
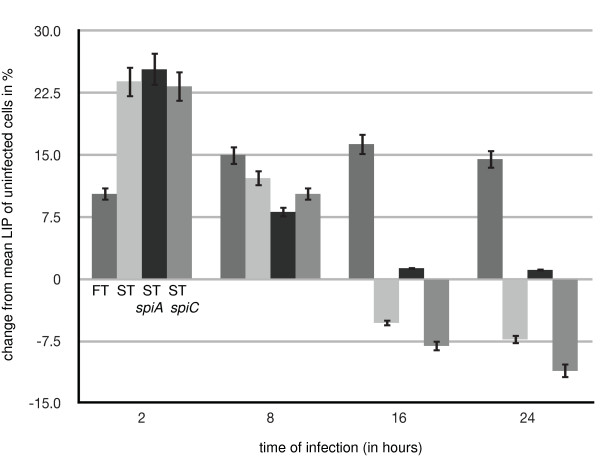
**Labile iron pool in macrophages during infection with *Francisella *and *Salmonella***. RAW264.7 macrophages were infected for 2 h, 8 h, 16 h, and 24 h with wild *Francisella *(FT), wild-type *Salmonella *(ST), *spiA Salmonella *(ST/spiA), or *spiC Salmonella *(ST/spiC). Labile iron pool was determined with the calcein method as described in detail in Materials and Methods. Measurements were in arbitrary fluorescence units standardized to uninfected samples. Data shown are the deviation in percentage from uninfected samples from triplicate experiments. Results are expressed as means +/- 1 standard error of mean (SEM).

While increased expression of TfR1 leads to an increase in the labile iron pool when exposed to iron-loaded transferrin, the overall labile iron pool (LIP) of the host cell can be affected in many different ways during infection. We therefore assessed the LIP during infection with *Francisella *by using the calcein method as described earlier [29] and compared it to the LIP during infection with *Salmonella*. After two hours of infection with *Francisella *and *Salmonella *there was a 10-25% increase in the labile iron pool (Figure 5; p = 0.01 for *Francisella*, p = 0.002 for *Salmonella*). Over the next twenty-two hours, macrophages infected with *Francisella *maintained an increased iron pool (Figure 5; p = 0.008 for 8 h, p = 0.002 for 16 h, and p = 0.005 for 24 h), whereas those infected with *Salmonella *showed a persistently decreasing iron pool (Figure 5; p = 0.002 for 8 h, p = 0.04 for 16 h, and p = 0.03 for 24 h).

We also measured changes in the labile iron pool during infection with two isogenic mutant *Salmonella *strains, *spiA *and *spiC*, which have intracellular trafficking deficits associated with reduced intracellular proliferation and avirulence in mice. These strains carry two different deletions in the SPI-2 type III secretion system (*spiA *and *spiC*) [32,33]. The rationale for using these strains in our experiments was to investigate if different subcellular localizations of a given pathogen can lead to different patterns in iron acquisition. After two hours of infection, the labile iron pool was increased similar to macrophages infected with wild-type *Salmonella *(Figure 5; p = 0.001 for *spiA Salmonella*, p = 0.002 for *spiC Salmonella*). After twenty-four hours, *spiC Salmonella *gradually decreased the iron pool similar to infection with wild type (Figure 5; p = 0.02 for 8 h, p = 0.02 for 16 h, p = 0.001 for 24 h). In contrast, the labile iron pool initially decreased and then remained unchanged during infection with *spiA Salmonella *(Figure 5; p = 0.02 for 8 h, p = 0.45 for 16 h, p = 0.56 for 24 h).

### Iron-related gene expression in macrophages infected with Salmonella or Francisella

Acquisition of iron through TfR1 requires expression of accessory gene products (Steap3, Dmt1) and can be countered by increased iron export (Fpn1) or scavenging of iron by the lipocalin system (Lcn2, LcnR). Induction of innate immune responses during infection can modulate iron homeostasis pathways through induction of hepcidin (Hamp1) and Lcn2. The expression of such genes and selected other genes that are involved in the homeostasis of host cell iron levels were investigated by real-time RT-PCR during infection with *Francisella *and compared to the expression profile of host cells during infection with *Salmonella*.

**Figure 6:**
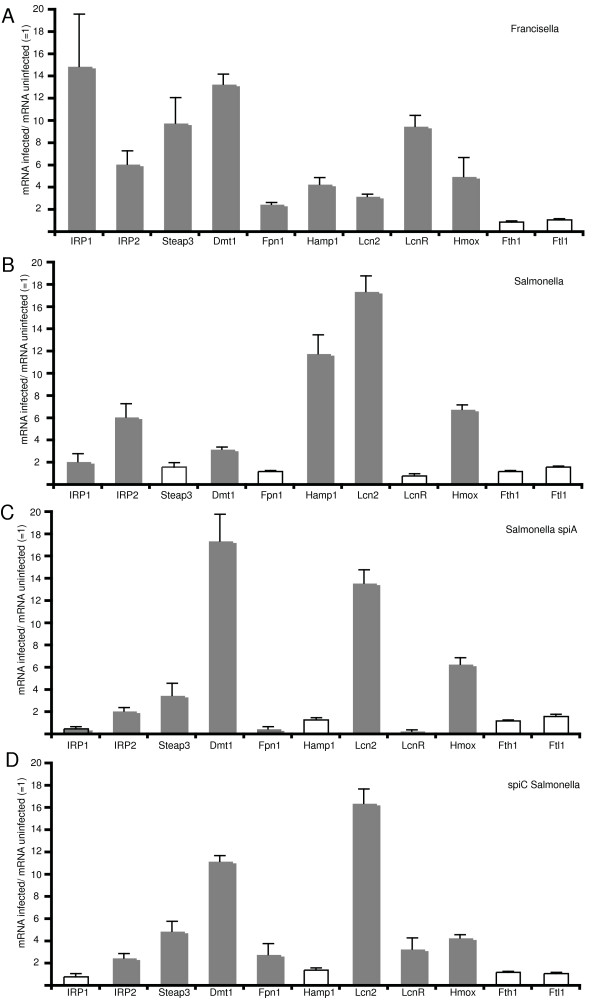
**Expression of genes involved in iron homeostasis during infection with *Francisella *or *Salmonella***. RAW264.7 macrophages were infected for 24 h with wild-type *Francisella *(A), wild type *Salmonella *(B), *spiC Salmonella *(C), or *spiA Salmonella *(D). Quantitative mRNA levels were determined by quantitative light cycler PCR for: iron-regulatory protein 1 (IRP1), iron regulatory protein 2 (IRP2), ferrireductase (Steap3), transmembrane iron transporter (Dmt1), lipocalin (Lcn2), lipocalin receptor (LcnR), ferroportin (Fpn1), antimicrobial peptide hepcidin (Hamp1), heme oxygenase (Hmox1), ferritin heavy chain 1(Fth1), ferritin light chain 1 (Ftl1), and ferritin light chain 2 (Ftl2). Measurements were standardized to GAPDH-mRNA levels for each experiment. Values shown represent the ratio of mRNA for a given gene in infected cells divided by the mRNA level in uninfected cells (mRNA infected/mRNA uninfected). Statistically significant expression data are shown by solid bars (Student's t-test, p < 0.05 is considered as significant; individual p-values are given in the text). Results from n = 6 experiments are expressed as means +/- 1 standard error of mean (SEM).

There are two main eukaryotic iron-regulatory proteins, IRP1 and IRP2, which sense changes in the labile iron pool and secondary signals associated with redox active species. They both act post-translationally by stabilizing their respective target mRNA and by affecting initiation of translation. While expression of IRP-2 is increased by *Salmonella *and *Francisella *(p = 0.003 and p = 0.002 respectively), IRP1 is strongly induced only in *Francisella*-infected cells (Figure 6A and 6B; p = 0.0001 for *Francisella*, p = 0.02 for *Salmonella*).

After uptake of iron via TfR1 and acidity-triggered release into the vesicle, ferric iron needs to be reduced, which is accomplished by the ferrireductase Steap3 [34]. After reduction, ferrous iron is transported into the cytosol by Dmt1 or functional Nramp1 [35,36]. There is a fivefold higher induction of Steap3 and Dmt1 during infection with *Francisella *(p = 0.0001) when compared to infection with wild-type *Salmonella *(p = 0.67) (Figure 6A and 6B).

Infected host cells can restrict the intracellular iron pool available for intracellular parasites by transporting iron out of the cells via ferroportin 1 (Fpn1), a transmembrane iron efflux protein [37]. While Fpn1 is increased 2.5-fold in macrophages infected with *Francisella *(p = 0.02), there is no change during infection with *Salmonella *(p = 0.46) (Figure 5A and 5B).

During infection with bacteria, hepatocytes secrete the antimicrobial peptide hepcidin (Hamp1), which binds to ferroportin on macrophages (and other cell types). This leads to internalization and degradation of ferroportin and entrapment of iron inside the cell. It was also shown recently that hepcidin is induced in myeloid cells through the TLR-4 pathway and regulates ferroportin levels at the transcriptional and post-translational level [38]. Hepcidin thus effectively reduces iron efflux [39-41]. There is a two-fold stronger induction of hepcidin during infection with *Salmonella *when compared to infection with *Francisella *(Figure 6A and 6B; p = 0.001 and p = 0.01 respectively). This might be explained by *Francisella *LPS preferentially stimulating the TLR-2 pathway, while *Salmonella *LPS induces the TLR-4 pathway [42].

The lipocalin system provides the host with another way of scavenging iron or withholding it from bacteria [43]. The host protein lipocalin (Lcn2) can interact with bacterial siderophores and has now been recognized as an important arm of the innate immune response after its production is stimulated by recognition of bacteria through TLR-4 pathways [44]. Lcn2 is induced twofold in cells infected with *Francisella *(p = 0.01), but more than 15-fold when cells are infected with *Salmonella *(p = 0.002). This might again be expected because of the strong induction of the TLR-4 pathway by *Salmonella *in comparison to the preferred TLR-2 induction by *Francisella*. *Salmonella*, however, do not raise mRNA levels for the lipocalin receptor (LcnR), which are significantly increased in *Francisella*-infected macrophages (Figure 6A and 6B).

Heme oxygenase (HO-1, Hmox1) catalyzes the conversion of heme to biliverdin, iron, and carbon monoxide. In macrophages it has an important antioxidative protective function, presumably by reducing pro-oxidant or pro-apoptotic hemoproteins [45,46]. Not unexpectedly, the mRNA level for Hmox1 is increased in macrophages infected by *Francisella *and *Salmonella *(Figure 6A and 6B; p = 0.002 and p = 0.002 respectively).

None of the components of the ferritin iron storage system are affected by infection with *Salmonella *or *Francisella *as measured by determining the expression of Fth1 and Ftl1 (Figure 6A and 6B; p = 0.91 and p = 0.90 for *Francisella *and p = 0.88 and p = 0.78 for *Salmonella*).

These gene-expression data suggest that *Francisella *drives a more active transferrin-mediated iron uptake program than *Salmonella*. Increased mRNA levels for IRP1 and IRP2 maintain increased translational levels for TfR1. Induction of genes required for transfer of iron to the cytosol via Dmt1 and Steap3 support the TfR1-mediated import route. Preferential induction of the TLR-4 pathway by *Salmonella *leads to a strong induction of hepcidin and lipocalin.

We further sought to characterize the expression profile of these iron-homoestasis-related genes in the *spiC *and *spiA Salmonella *mutants, which lead to variable alterations in the LIP (Figure 5). Both mutant strains have a higher increase in the Steap3/DMT1 genes than wild-type *Salmonella *(p = 0.01 and p = 0.001 for *spiA Salmonella*, and p = 0.01 and p = 0.003 for *spiC Salmonella*), while the induction of the iron-regulatory proteins IRP1 and IRP2 are lower (p = 0.02 for IRP1 and p = 0.02 for IRP2 in *spiA Salmonella*; p = 0.35 for IRP1 and p = 0.02 for IRP2 in *spiC Salmonella*). While TLR-4 driven induction of lipocalin is maintained in the mutant strains (p = 0.002 for *spiA *and p = 0.001 for *spiC Salmonella*), there is no induction of hepcidin (p = 0.89 and p = 0.78 respectively). The iron exporter Fpn1 is increased threefold in the *spiC *mutant (p = 0.01), while there is no increase in the *spiA *mutant (p = 0.78) (Figure 6C and 6D). This might be one possible explanation for the decrease in the labile iron pool in the *spiC *mutant in comparison to the *spiA *mutant (Figure 5). These findings suggest that distinct bacterial gene mutations with associated aberrant intracellular trafficking can affect the expression of iron homeostasis genes in the host cell.

## Discussion

We have characterized two different phenotypes of host cell and intracellular bacterial pathogen behavior in relation to host cell iron metabolism and bacterial iron requirements. *Francisella *drives an active iron acquisition program through the transferrin receptor TfR1 with a sustained increase in the host cell labile iron pool. Since *Francisella *depends on expression of TfR1 for intracellular survival, it might need an increased host cell iron level for its own metabolism and might be able to efficiently counteract increased production of host cell reactive redox species. *Salmonella*, on the other hand, does not require TfR1 for growth inside its host cell, lacks a strong induction of gene products aimed at facilitating iron import via TfR1, and negotiates a decreased iron level in the host cell. This might be explained by *Salmonella*'s intracellular localization within an endosomal structure or perhaps by more efficient iron acquisition strategies. The distinction of these two phenotypes will allow further characterization and understanding of eukaryotic iron metabolism and its modulation by intracellular bacteria.

*Francisella *enters macrophages inside an early endosome, from which it later escapes into the cell cytosol [13]. We have provided corroborating evidence that entry occurs in an early endosome with recruitment of TfR1 and Rab5, but no acquisition of Rab7, which is a prerequisite for further maturation in the phagolysosomal trafficking pathway. In this study we have demonstrated a very early co-localization of TfR1 and *Francisella *at the cell surface. This suggests that TfR1 is recruited during the entry process rather than by successive fusion of *Francisella*-containing vesicles with TfR1-carrying endosomes. Such a process differs from *M. tuberculosis*-containing vesicles, which recruit TfR through endosome fusions during infection of the host cell [11].

Increased expression of the transferrin receptor has been shown previously during infection with *Ehrlichia*, *Chlamydia*, and *Coxiella*, while reduced or unaltered expression was observed during infection with *Salmonella *and *Legionella *[28,47] as a means of host defense by restricting the iron available for the invading pathogen. In fact, decreased expression of TfR1 in a patient due to a chronic inflammatory condition (with increased IFN-γ production) proved non-permissive for infection with *Legionella *[48]. Infection with *Ehrlichia chafeensis *and *E. sennetsu *changes the binding affinities for IRP-1 during the first hours of infection with a concomitant increase in levels of transferrin receptor. This is followed by a response at the transcriptional level of transferrin receptor mRNA at 24 h of infection [10]. Similar to our observations with *Francisella*, these effects require viable *Ehrlichia *and cannot be caused by the Human Granulocytic Ehrlichiosis Agent. While *Francisella *shows a very early and intense colocalization with TfR and then escapes from the vesicle, *Ehrlichia *remains in a membranous compartment, which is characterized by Rab5 and EEA1 and only over time recruits TfR1 [49]. While our studies did not address the mechanisms by which *Francisella *increases the expression of TfR1, we speculate that a disruption of the host cell home iron homeostasis system causes the cell to sense a low iron balance with subsequent initiation of an active iron acquisition program. We cannot rule out that some bacterial product directly or indirectly through intermediates of inflammation affects IRP-1 binding affinities or that other yet uncharacterized cytokine activation pathway triggered by the infection play a role.

While it is known that TfR1 transports Fe-loaded transferrin to the bacterium-containing vesicle, it is not at all clear that iron delivered in this way can be utilized by bacteria. For *M. tuberculosis *it could be demonstrated that Fe delivered by transferrin can be utilized [50]. Based on the kinetics of Fe delivery it was calculated, however, that at least a portion of the Fe delivered by transferrin is first delivered to the cytosol, presumably through the action of DMT1 [51]. While siderophores clearly play a role, it could also be demonstrated that these exochelins cannot directly remove Fe from transferrin [52]. It has also not been shown if such siderophores could actually transverse the endosome membrane. Our data demonstrate that *Francisella *actively upregulates TfR1, which leads to an improved delivery of iron into the labile intracellular iron pool. In contrast to *Salmonella*, *Francisella *also drives an active iron acquisition program with upregulation of accessory iron metabolic genes such as the iron transporter Dmt1 and the ferrireductase Steap3, which all serve to promote the import of iron from TfR1 to the cytosol. We propose that *Francisella *can directly exploit the concomitant increase in LIP during infection, whereas such an increase would be of little benefit to *Salmonella *with a preferentially endosomal location.

A recent study has examined the expression profile of selected iron-homeiostasis genes and iron-loading of ferritin in murine macrophages during infection with *Salmonella *[28]. While their findings agree with ours with regard to the upregulation of Lcn2, Hmox1, and Hamp, the authors could not find a significant increase in Dmt1, but did see an increase in Fpn1. This correlated with their observation of increased iron efflux from infected cells and decreased iron content of ferritin. Some of the differences between our data and theirs might be explained by their use of a particular *Salmonella *strain (C5RP4). Of particular interest in this context is that the *spiC Salmonella *mutant strain used in our studies behaves quite similiar to the C5RP4 strain by demonstrating an increase in Fpn1 (Figure 6D). It is thus conceivable that the *Salmonella *strain employed by Nairz and colleagues carries distinct uncharacterized gene mutations or phenotypes. Our assessment of the labile iron pool after infection with *Salmonella *after 24 h shows a decrease (Figure 5) and agrees with the findings reported by Nairz [28].

## Conclusions

Iron acquisition and utilization by microbes is of critical importance for bacterial pathogenesis. Defects in the bacterium's ability to efficiently scavenge iron and use it in its metabolism usually lead to avirulence. However, little is known how bacteria might modulate the iron handling properties of their host cells.

We identified two distinct iron-handling scenarios for two different bacterial pathogens. *Francisella tularensis *drives an active iron acquisition program via the TfR1 pathway program with induction of ferrireductase (Steap3), iron membrane transporter Dmt1, and iron regulatory proteins IRP1 and IRP2, which is associated with a sustained increase of the labile iron pool inside the macrophage. Expression of TfR1 is critical for *Francisella's *intracellular proliferation. This contrasts with infection of macrophages by wild-type *Salmonella typhimurium*, which does not require expression of TfR1 for successful intracellular survival. Macrophages infected with *Salmonella *lack significant induction of Dmt1, Steap3, and IRP1, and maintain their labile iron pool at normal levels.

## Methods

### Bacterial strains, cell lines, growth conditions, and plasmids

*Francisella tularensis subspecies holarctica *vaccine strain (*F. tularensis *LVS, army lot 11) was generously provided to us by Dr. Karen Elkins (FDA). *F. tularensis *LVS was transformed with plasmid pFNLTP6 *gro-gfp *to produce a *Francisella *strain constitutively expressing green fluorescent protein (SD833). Wild-type *Salmonella *strain ATCC 14028 was used. *Salmonella *mutant strains *spiC::kan *(EG10128) and *spiA::kan *(EG5793) are isogenic derivatives [32]. *Francisella *was grown on chocolate II agar enriched with IsoVitaleX (BD Biosciences, San Jose, CA) for 40-48 hrs at 37°C. For liquid medium, we used Mueller-Hinton broth supplemented with IsoVitaleX. *Salmonella *strains and *E.coli *XL-1 were grown at 37°C with shaking in LB broth without glucose or on LB plates [53]. When indicated antibiotics were present (in μg/ml) at: kanamycin, 50; chloramphenicol, 50; for *Francisella*, kanamycin was used at 10 μg/ml.

RAW264.7 murine macrophages were obtained from ATCC (TIB-71). Dulbecco's Modification of Eagle's Medium (DMEM; Cellgro) was supplemented with 10% fetal bovine serum (Hyclone, not heat-inactivated) and penicillin (100 I.U./ml) and streptomycin (100 μg/ml). When cells were used for *Francisella *infection assays, no antibiotics were added 24 h prior to infection. Cells were grown at 37°C and 5%CO2.

A shuttle plasmid which encodes Gfp under the control of the *groE *promoter (pFNLTP6 *gro-gfp*) was kindly provided to us by Dr. Zahrt [54]. It carries a kanamycin antibiotic resistance marker.

### Infection Assay

Several colonies of F. *tularensis *LVS were collected, washed twice with sterile phosphate buffer at pH 7.0 (PBS, Mediatech Inc #46-013-CM), and dispersed in cell culture complete medium for 15 minutes. Multiplicity of infection was adjusted to 10 using a standardized calibration curve of OD_600_/colony-forming units (cfu). Bacteria were added to host cells at 60-80% confluency in 12-well dishes. At a given timepoint after the infection, host cells were washed repeatedly with warm PBS. If indicated, remaining extra-cellular bacteria were killed by the addition of 10 μg/ml of gentamicin to DMEM (37°C, 5% CO2) for 60 minutes. Time points given in the text for infection include this 60 minute time period of culture in the presence of gentamicin, except when infected cells were processed for immunostaining. Gentamicin was removed by washing in DMEM. Infected cells were resuspended in complete tissue culture medium without addition of antibiotics. After a given time of infection, cells were lyzed in 0.5% N-octyl β-glucopyranoside (Bioscience). Serial dilutions of cell lysates were plated on Chocolate II agar and incubated at 37°C for at two days. Infection with *Salmonella *was performed as described [55]. Comparison of infection results were analyzed by the Student's t-test, p < 0.05 was considered significant.

### Immunostaining

Macrophage cell lines were grown on sterile coverslips in Petri dishes (6- or 12-well plates). Cells were infected with *Francisella *as described above, except that the step of killing extracellular bacteria with gentamicin was substituted by washing of adherent cells with DMEM three times. At indicated time points, cells on coverslips were fixed in 4% paraformaldehyde solution (Polysciences, #18814) for 10 minutes, washed with PBS and permeabilized in 0.1% Triton × 100 (Shelton Scientific IB07100) in PBS for 15 minutes. Reaction with antisera was performed in 0.05% TWEEN20/PBS for one hour at room temperature. Stained and dried coverslips were mounted on glass slide using Gold antifade medium (Invitrogen, #P36930) and sealed with nail polish

Antiserum to TfR1 was goat polyclonal IgG (SantaCruz sc 7087), to Rab5, rabbit polyclonal IgG (Santa Cruz SC-309) and to Rab7, goat polyclonal IgG (SC11303). Antibodies were used at a dilution of 1:500. Visualization was with staining with a goat-anti-rabbit or rabbit-anti-goat IgG conjugated to Alexa 594 (Invitrogen).

### Microscopy

A Leica AOBS laser scanning microscope was used for all fluorescence microscopy. Images were acquired using Leica software. Analyses of images was with Volocity software (Volocity 4.1 Imporvision Inc., Lexington, MA). Overlap of individual fluorescence pixels from separate channels for each optical plane was determined with the Volocity 4.1 colocalization module. When results were quantified, 100 cells from randomly selected fields were evaluated. All cells found in a given field were analyzed, except for cells with obvious signs of cell death (detachment, ballooning), which were excluded (in general < 5%). Results are reported as the percentage of 100 cells analyzed. Groupwise comparison was made by the Student's t-test, p < 0.05 was considered significant.

### RNA interference

Two siRNAs for TfR1 (Tfrc_4 (TACCCATGACGTTGAATTGAA), and Tfrc_1 (ATCGTTAGTATCTAACATGAA)) were designed using proprietary software and synthesized. Both had 3' modifications with Alexa Fluor 555. Transfection of macrophages was accomplished with Lipofectamine 2000 according to the manufacturer's instruction. Only Tfrc1 had significant activity (data not shown) and was used for all further studies

### Real-time RT-PCR

Total RNA was isolated and digested with DNAse using the Microto-Midi Total RNA Purification System (Invitrogen, catalog no. 12183-018) according to the product instructions. RNA concentrations were determined by a RiboGreen assay (Molecular Probes, Carlsbad, CA; catalog no. R11490). Primer design was performed with the eXpress Profiling Suite software (Beckman) and mRNA sequences from the GenBank database. Uniqueness and specificity of each primer was verified using the Basic Local Alignment Search Tool http://www.ncbi.nlm.nih.gov/blast returning Genbank accession numbers. Primers are listed in Table 1.

**Table 1:** Primers used for real-time RT PCR

Gene	Accession number	Forward primer (5' → 3')	Reverse Primer (5' → 3')
GAPDH	NM_008084	AGGTGACACTATAGAATACCCACTAACATCAAATGGGG	GTACGACTCACTATAGGGACCTTCCACAATGCCAAAGTT
IRP1	NM_007386	AGGTGACACTATAGAATAACTTTGAAAGCTGCCTTGGA	GTACGACTCACTATAGGGACTCCACTTCCAGGAGACAGG
IRP2	NM_022655	AGGTGACACTATAGAATATGAAGAAACGGACCTGCTCT	GTACGACTCACTATAGGGAGCTCACATCCAACCACCTCT
TfR1	BC054522	AGGTGACACTATAGAATATGCAGAAAAGGTTGCAAATG	GTACGACTCACTATAGGGATGAGCATGTCCAAAGAGTGC
Dmt1	NM_008732	AGGTGACACTATAGAATAGCCAGCCAGTAAGTTCAAGG	GTACGACTCACTATAGGGAGCTGTCCAGGAAGACCTGAG
LcnR	NM_021551	AGGTGACACTATAGAATAGCAAGGCTACCCCATACAAA	GTACGACTCACTATAGGGAAAGAGCGAGGTCTGGGAAAT
Lcn2	NM_008491	AGGTGACACTATAGAATACTGAATGGGTGGTGAGTGTG	GTACGACTCACTATAGGGATATTCAGCAGAAAGGGGACG
Steap3	BC037435	AGGTGACACTATAGAATACTCTCTGTGCAGTCTCGCTG	GTACGACTCACTATAGGGATGCAGAGATGACGTTGAAGG
Hmox1	NM_010442	AGGTGACACTATAGAATACCTCACTGGCAGGAAATCAT	GTACGACTCACTATAGGGACCAGAGTGTTCATTCGAGCA
Fpn1	AF226613	AGGTGACACTATAGAATATGCCTTAGTTGTCCTTTGGG	GTACGACTCACTATAGGGAGTGGAGAGAGAGTGGCCAAG
Hamp1	NM_032541	AGGTGACACTATAGAATAGAGAGACACCAACTTCCCCA	GTACGACTCACTATAGGGATCAGGATGTGGCTCTAGGCT
Ftl1	NM_010240	AGGTGACACTATAGAATAAAGATGGGCAACCATCTGAC	GTACGACTCACTATAGGGAGCCTCCTAGTCGTGCTTGAG
Fth1	NM_010239	AGGTGACACTATAGAATACTCATGAGGAGAGGGAGCAT	GTACGACTCACTATAGGGAGTGCACACTCCATTGCATTC

The reverse transcription reactions were carried out with 20 units of Moloney Murine Leukemia Virus (MMuLV) reverse transcriptase (Fisher Scientific, catalog no. BP3208-1), 20 units RNase inhibitor (Fisher Scientific, catalog no. BP3225-1), RT-PCR buffer containing 10 mM Tris-HCl and 50 mM KCl; 2.5 mM MgCl_2_; 10 mM dithiothreitol; and 1 mM of each dNTP. The concentration of each reverse primer was 5 μM. 100 ng of total RNA from each sample was reverse transcribed using reverse primers. The reverse transcription reactions were incubated for 1 min at 48°C, 5 min at 37°C, 60 min at 42°C, and then 5 min at 95°C.

Real-time RT-PCR was based on the high affinity, double-stranded DNA-binding dye SYBR Green using a Bio-Rad IQ SYBR Green Supermix according to manufacturer's instructions. A total of 2 μl of cDNA was used in the qPCR reactions (1 × SYBR green PCR master mix, 500 nM gene specific forward and reverse primers). All qPCR reactions started with 2 min at 95°C followed by 40 cycles of 15 s at 94°C and 20 s at 55°C and 30 s at 72°C in an Applied Biosystems 7900HT Fast Real-Time PCR System. Differences in mRNA concentrations were quantified by the cycles to fluorescence midpoint cycle threshold calculation (2- [ΔCt experimental gene- ΔCt housekeeping gene]), using GAPDH as the housekeeping gene. Comparisons between two groups were performed with Statview 9.1.3 statistical analysis software using the Student's t-test. P < 0.05 was considered statistically significant. All results are expressed as means +/- 1 standard error of the mean (SEM).

### Determination of the labile iron pool with calcein-AM

Relative alterations in the levels of "labile iron pool" (LIP) by the upregulated transferrin receptors during the infection of *Francisella *in macrophages were determined with the fluorescent metalosensor calcein-AM [29,56]. Infection of RAW 264.7 macrophages with *Francisella *was carried at the MOI of 10. After 1 hr and 24 hrs of infection cells were detached from plates using a rubber policeman and used in suspension. Uninfected controls were maintained as well.

A total of 5.5 × 10^6 ^infected macrophages were washed three times with warm DMEM. The cells were suspended in DMEM and then incubated with 0.125 μM calcein-AM (Invitrogen, #C3100MP) for 10 min at 37°C. After three washes with warm PBS to remove unbound calcein, the cells were resuspended in warm PBS. 200 μl (5 × 10^4^) of calcein-loaded cells were suspended in a 5 × 13 mm glass cuvette (Wheaton, Milleville, NJ #225350). Fluorescence was monitored on a TD700 Fluorimeter (Turner Designs, Sunnyvale, CA) (488-nm excitation and 517-nm emission) at 37°C. After stabilization of the signal, 10 μg/ml of holo-transferrin (Sigma, #T1283) was added to measure the changes in the intracellular calcein-bound iron pool of the infected cells. Fluorescent units were measured at one-second intervals. For comparative determination of the total cellular LIP, infected and uninfected macrophages were loaded with calcein-AM as above. Fluorescence (F) was measured exactly ten minutes after loading with calcein-AM in a TD700 fluorimeter. A cell permeable Fe-chelator was added as described (16, [29]. Dequenched fluorescence (Δ F) was again determined 5 minutes after addition of deferrioxamine. Both values, F and Δ F, showed a linear correlation and represent the relative total macrophage LIP.

## Authors' contributions

XP and BT performed experiments and analyzed data, SD designed experiments, analyzed data, and drafted manuscript, EH provided critical guidance, insights, and suggestions. All authors read and approved the final manuscript.

## References

[B1] Radtke AL O'Riordan MX Intracellular innate resistance to bacterial pathogens Cell Microbiol 2006 8 1720 1729 10.1111/j.1462-5822.2006.00795.x 16939532

[B2] Paradkar P De Domenico I Durchfort N Zohn I Kaplan J Ward DM Iron-depletion limits intracellular bacterial growth in macrophages Blood 2008 112 866 874 10.1182/blood-2007-12-126854 18369153 PMC2481528

[B3] Collins HL The role of iron in infections with intracellular bacteria Immunol Lett 2003 85 193 195 10.1016/S0165-2478(02)00229-8 12527227

[B4] Chlosta S Fishman DS Harrington L Johnson EE Knutson MD Wessling-Resnick M Cherayil BJ The iron efflux protein ferroportin regulates the intracellular growth of Salmonella enterica Infect Immun 2006 74 3065 3067 10.1128/IAI.74.5.3065-3067.2006 16622252 PMC1459754

[B5] Bullen JJ Rogers HJ Spalding PB Ward CG Natural resistance, iron and infection: a challenge for clinical medicine J Med Microbiol 2006 55 251 258 10.1099/jmm.0.46386-0 16476787

[B6] Schaible UE Kaufmann SH Iron and microbial infection Nat Rev Microbiol 2004 2 946 953 10.1038/nrmicro1046 15550940

[B7] Kehrer JP The Haber-Weiss reaction and mechanisms of toxicity Toxicology 2000 149 43 50 10.1016/S0300-483X(00)00231-6 10963860

[B8] Theurl I Fritsche G Ludwiczek S Garimorth K Bellmann-Weiler R Weiss G The macrophage: a cellular factory at the interphase between iron and immunity for the control of infections Biometals 2005 18 359 367 10.1007/s10534-005-3710-1 16158228

[B9] Howe D Mallavia LP Coxiella burnetii infection increases transferrin receptors on J774A. 1 cells Infect Immun 1999 67 3236 3241 10377096 10.1128/iai.67.7.3236-3241.1999 PMC116501

[B10] Barnewall RE Ohashi N Rikihisa Y Ehrlichia chaffeensis and E. sennetsu, but not the human granulocytic ehrlichiosis agent, colocalize with transferrin receptor and up-regulate transferrin receptor mRNA by activating iron-responsive protein 1 Infect Immun 1999 67 2258 2265 10225882 10.1128/iai.67.5.2258-2265.1999 PMC115965

[B11] Clemens DL Horwitz MA The Mycobacterium tuberculosis phagosome interacts with early endosomes and is accessible to exogenously administered transferrin J Exp Med 1996 184 1349 1355 10.1084/jem.184.4.1349 8879207 PMC2192850

[B12] Steele-Mortimer O The Salmonella-containing vacuole-Moving with the times Curr Opin Microbiol 2008 11 38 45 10.1016/j.mib.2008.01.002 18304858 PMC2577838

[B13] Clemens DL Lee BY Horwitz MA Virulent and avirulent strains of Francisella tularensis prevent acidification and maturation of their phagosomes and escape into the cytoplasm in human macrophages Infect Immun 2004 72 3204 3217 10.1128/IAI.72.6.3204-3217.2004 15155622 PMC415696

[B14] Deng K Blick RJ Liu W Hansen EJ Identification of Francisella tularensis genes affected by iron limitation Infect Immun 2006 74 4224 4236 10.1128/IAI.01975-05 16790797 PMC1489736

[B15] Sullivan JT Jeffery EF Shannon JD Ramakrishnan G Characterization of the siderophore of Francisella tularensis and role of fslA in siderophore production J Bacteriol 2006 188 3785 3795 10.1128/JB.00027-06 16707671 PMC1482922

[B16] Su J Yang J Zhao D Kawula TH Banas JA Zhang JR Genome-wide identification of Francisella tularensis virulence determinants Infect Immun 2007 75 3089 3101 10.1128/IAI.01865-06 17420240 PMC1932872

[B17] Weiss DS Brotcke A Henry T Margolis JJ Chan K Monack DM In vivo negative selection screen identifies genes required for Francisella virulence Proc Natl Acad Sci USA 2007 104 6037 6042 10.1073/pnas.0609675104 17389372 PMC1832217

[B18] Lenco J Hubalek M Larsson P Fucikova A Brychta M Macela A Stulik J Proteomics analysis of the Francisella tularensis LVS response to iron restriction: induction of the F. tularensis pathogenicity island proteins IglABC FEMS Microbiol Lett 2007 269 11 21 10.1111/j.1574-6968.2006.00595.x 17227466

[B19] Pekarek RS Bostian KA Bartelloni PJ Calia FM Beisel WR The effects of Francisella tularensis infection on iron metabolism in man Am J Med Sci 1969 258 14 25 10.1097/00000441-196907000-00003 4894492

[B20] McKenna WR Mickelsen PA Sparling PF Dyer DW Iron uptake from lactoferrin and transferrin by Neisseria gonorrhoeae Infect Immun 1988 56 785 791 3126143 10.1128/iai.56.4.785-791.1988 PMC259371

[B21] Ratledge C Dover LG Iron metabolism in pathogenic bacteria Annu Rev Microbiol 2000 54 881 941 10.1146/annurev.micro.54.1.881 11018148

[B22] Braun V Iron uptake mechanisms and their regulation in pathogenic bacteria Int J Med Microbiol 2001 291 67 79 10.1078/1438-4221-00103 11437341

[B23] Lindgren H Honn M Golovlev I Kadzhaev K Conlan W Sjostedt A The 58-kilodalton major virulence factor of Francisella tularensis is required for efficient utilization of iron Infect Immun 2009 77 4429 4436 10.1128/IAI.00702-09 19651867 PMC2747937

[B24] Rohmer L Brittnacher M Svensson K Buckley D Haugen E Zhou Y Chang J Levy R Hayden H Forsman M Olson M Johansson A Kaul R Miller SI Potential source of Francisella tularensis live vaccine strain attenuation determined by genome comparison Infect Immun 2006 74 6895 6906 10.1128/IAI.01006-06 17000723 PMC1698093

[B25] Hashim S Mukherjee K Raje M Basu SK Mukhopadhyay A Live Salmonella modulate expression of Rab proteins to persist in a specialized compartment and escape transport to lysosomes J Biol Chem 2000 275 16281 16288 10.1074/jbc.275.21.16281 10821869

[B26] Steele-Mortimer O Meresse S Gorvel JP Toh BH Finlay BB Biogenesis of Salmonella typhimurium-containing vacuoles in epithelial cells involves interactions with the early endocytic pathway Cell Microbiol 1999 1 33 49 10.1046/j.1462-5822.1999.00003.x 11207539

[B27] Simpson JC Jones AT Early endocytic Rabs: functional prediction to functional characterization Biochem Soc Symp 2005 99 108 15649134 10.1042/bss0720099

[B28] Nairz M Theurl I Ludwiczek S Theurl M Mair SM Fritsche G Weiss G The co-ordinated regulation of iron homeostasis in murine macrophages limits the availability of iron for intracellular Salmonella typhimurium Cell Microbiol 2007 9 2126 2140 10.1111/j.1462-5822.2007.00942.x 17466014

[B29] Kakhlon O Cabantchik ZI The labile iron pool: characterization, measurement, and participation in cellular processes(1) Free Radic Biol Med 2002 33 1037 1046 10.1016/S0891-5849(02)01006-7 12374615

[B30] Rothman RJ Serroni A Farber JL Cellular pool of transient ferric iron, chelatable by deferoxamine and distinct from ferritin, that is involved in oxidative cell injury Mol Pharmacol 1992 42 703 710 1435746

[B31] Breuer W Epsztejn S Cabantchik ZI Iron acquired from transferrin by K562 cells is delivered into a cytoplasmic pool of chelatable iron(II) J Biol Chem 1995 270 24209 24215 10.1074/jbc.270.33.19330 7592626

[B32] Uchiya K Barbieri MA Funato K Shah AH Stahl PD Groisman EA A Salmonella virulence protein that inhibits cellular trafficking Embo J 1999 18 3924 3933 10.1093/emboj/18.14.3924 10406797 PMC1171468

[B33] Ochman H Soncini FC Solomon F Groisman EA Identification of a pathogenicity island required for Salmonella survival in host cells Proc Natl Acad Sci USA 1996 93 7800 7804 10.1073/pnas.93.15.7800 8755556 PMC38828

[B34] Ohgami RS Campagna DR McDonald A Fleming MD The Steap proteins are metalloreductases Blood 2006 108 1388 1394 10.1182/blood-2006-02-003681 16609065 PMC1785011

[B35] Andrews NC Schmidt PJ Iron homeostasis Annu Rev Physiol 2007 69 69 85 10.1146/annurev.physiol.69.031905.164337 17014365

[B36] Hentze MW Muckenthaler MU Andrews NC Balancing acts: molecular control of mammalian iron metabolism Cell 2004 117 285 297 10.1016/S0092-8674(04)00343-5 15109490

[B37] Donovan A Brownlie A Zhou Y Shepard J Pratt SJ Moynihan J Paw BH Drejer A Barut B Zapata A Law TC Brugnara C Lux SE Pinkus GS Pinkus JL Kingsley PD Palis J Fleming MD Andrews NC Zon LI Positional cloning of zebrafish ferroportin1 identifies a conserved vertebrate iron exporter Nature 2000 403 776 781 10.1038/35001596 10693807

[B38] Peyssonnaux C Zinkernagel AS Datta V Lauth X Johnson RS Nizet V TLR4-dependent hepcidin expression by myeloid cells in response to bacterial pathogens Blood 2006 107 3727 3732 10.1182/blood-2005-06-2259 16391018 PMC1895778

[B39] Ludwiczek S Aigner E Theurl I Weiss G Cytokine-mediated regulation of iron transport in human monocytic cells Blood 2003 101 4148 4154 10.1182/blood-2002-08-2459 12522003

[B40] Nguyen NB Callaghan KD Ghio AJ Haile DJ Yang F Hepcidin expression and iron transport in alveolar macrophages Am J Physiol Lung Cell Mol Physiol 2006 291 L417 25 10.1152/ajplung.00484.2005 16648237

[B41] Nicolas G Viatte L Lou DQ Bennoun M Beaumont C Kahn A Andrews NC Vaulont S Constitutive hepcidin expression prevents iron overload in a mouse model of hemochromatosis Nat Genet 2003 34 97 101 10.1038/ng1150 12704388

[B42] Cole LE Elkins KL Michalek SM Qureshi N Eaton LJ Rallabhandi P Cuesta N Vogel SN Immunologic consequences of Francisella tularensis live vaccine strain infection: role of the innate immune response in infection and immunity J Immunol 2006 176 6888 6899 16709849 10.4049/jimmunol.176.11.6888

[B43] Devireddy LR Gazin C Zhu X Green MR A cell-surface receptor for lipocalin 24p3 selectively mediates apoptosis and iron uptake Cell 2005 123 1293 1305 10.1016/j.cell.2005.10.027 16377569

[B44] Flo TH Smith KD Sato S Rodriguez DJ Holmes MA Strong RK Akira S Aderem A Lipocalin 2 mediates an innate immune response to bacterial infection by sequestrating iron Nature 2004 432 917 921 10.1038/nature03104 15531878

[B45] Morse D Choi AM Heme oxygenase-1: from bench to bedside Am J Respir Crit Care Med 2005 172 660 670 10.1164/rccm.200404-465SO 15901614

[B46] Orozco LD Kapturczak MH Barajas B Wang X Weinstein MM Wong J Deshane J Bolisetty S Shaposhnik Z Shih DM Agarwal A Lusis AJ Araujo JA Heme oxygenase-1 expression in macrophages plays a beneficial role in atherosclerosis Circ Res 2007 100 1703 1711 10.1161/CIRCRESAHA.107.151720 17495224

[B47] Byrd TF Horwitz MA Interferon gamma-activated human monocytes downregulate transferrin receptors and inhibit the intracellular multiplication of Legionella pneumophila by limiting the availability of iron J Clin Invest 1989 83 1457 1465 10.1172/JCI114038 2496141 PMC303847

[B48] Byrd TF Horwitz MA Aberrantly low transferrin receptor expression on human monocytes is associated with nonpermissiveness for Legionella pneumophila growth J Infect Dis 2000 181 1394 1400 10.1086/315390 10762570

[B49] Barnewall RE Rikihisa Y Lee EH Ehrlichia chaffeensis inclusions are early endosomes which selectively accumulate transferrin receptor Infect Immun 1997 65 1455 1461 9119487 10.1128/iai.65.4.1455-1461.1997 PMC175153

[B50] Olakanmi O Britigan BE Schlesinger LS Gallium disrupts iron metabolism of mycobacteria residing within human macrophages Infect Immun 2000 68 5619 5627 10.1128/IAI.68.10.5619-5627.2000 10992462 PMC101514

[B51] Olakanmi O Schlesinger LS Ahmed A Britigan BE Intraphagosomal Mycobacterium tuberculosis acquires iron from both extracellular transferrin and intracellular iron pools. Impact of interferon-gamma and hemochromatosis J Biol Chem 2002 277 49727 49734 10.1074/jbc.M209768200 12399453

[B52] Gobin J Horwitz MA Exochelins of Mycobacterium tuberculosis remove iron from human iron-binding proteins and donate iron to mycobactins in the M. tuberculosis cell wall J Exp Med 1996 183 1527 1532 10.1084/jem.183.4.1527 8666910 PMC2192514

[B53] Miller JH 1972 Cold Spring Harbor, NY, USA: Cold Spring Harbor Laboratory

[B54] Maier TM Havig A Casey M Nano FE Frank DW Zahrt TC Construction and characterization of a highly efficient Francisella shuttle plasmid Appl Environ Microbiol 2004 70 7511 7519 10.1128/AEM.70.12.7511-7519.2004 15574954 PMC535190

[B55] Lee AH Papari M Daefler S Identification of a NIPSNAP homologue as host cell target for Salmonella virulence protein SpiC Cell Microbiol 2002 4 739 750 10.1046/j.1462-5822.2002.00225.x 12427096

[B56] Epsztejn S Kakhlon O Glickstein H Breuer W Cabantchik I Fluorescence analysis of the labile iron pool of mammalian cells Anal Biochem 1997 248 31 40 10.1006/abio.1997.2126 9177722

